# Spontaneous vocal coordination of vocalizations to water noise in rooks (*Corvus frugilegus*): An exploratory study

**DOI:** 10.1002/ece3.9791

**Published:** 2023-02-16

**Authors:** Maëlan Tomasek, Andrea Ravignani, Palmyre H. Boucherie, Sophie Van Meyel, Valérie Dufour

**Affiliations:** ^1^ Ecole Normale Supérieure de Lyon Lyon France; ^2^ UMR 7247, Physiologie de la reproduction et des comportements, INRAE, CNRS, IFCE Université de Tours Strasbourg France; ^3^ Comparative Bioacoustics Group Max Planck Institute for Psycholinguistics Nijmegen The Netherlands; ^4^ Center for Music in the Brain, Department of Clinical Medicine Aarhus University & The Royal Academy of Music Aarhus C Denmark; ^5^ Department of Cognitive Biology University of Vienna Vienna Austria; ^6^ Institut de Recherche sur la Biologie de l'Insecte, UMR 7261, CNRS University of Tours Tours France

**Keywords:** corvids, stimulation, temporal contingency, vocal control

## Abstract

The ability to control one's vocal production is a major advantage in acoustic communication. Yet, not all species have the same level of control over their vocal output. Several bird species can interrupt their song upon hearing an external stimulus, but there is no evidence how flexible this behavior is. Most research on corvids focuses on their cognitive abilities, but few studies explore their vocal aptitudes. Recent research shows that crows can be experimentally trained to vocalize in response to a brief visual stimulus. Our study investigated vocal control abilities with a more ecologically embedded approach in rooks. We show that two rooks could spontaneously coordinate their vocalizations to a long‐lasting stimulus (the sound of their small bathing pool being filled with a water hose), one of them adjusting roughly (in the second range) its vocalizations as the stimuli began and stopped. This exploratory study adds to the literature showing that corvids, a group of species capable of cognitive prowess, are indeed able to display good vocal control abilities.

## 1 INTRODUCTION

Vocal production is an important feature of animal communication and its functions are as diverse as territory defense, courtship, or exchange of information (Chen & Wiens, 2020). Vocal control is the ability to modify elements of an ongoing vocal output (Miller et al., 2003) and it undeniably confers benefits to birds as it enables them to adapt their response according to external stimuli. For example, birds can interrupt their vocalization upon hearing a predator approaching or match their neighbor's song when engaging in vocal competition (Beecher et al., 2000; Mougeot & Bretagnolle, 2000; Schmidt & Belinsky, 2013). This ability can include volitional processes, but also other types of involuntary processes like affective (emotional) processes (Hopf et al., 1992; Nieder & Mooney, 2019). Indeed, changes in the level of arousal can trigger the modulations of a call. The wide diversity of species using vocal communication also implies a broad spectrum of vocal control (Miller et al., 2003). Note that it seems unlikely that a species could have no control whatsoever over its vocal production. There should be some species with little control at one end of the spectrum, that is, the vocal output cannot be modulated once it has begun, whilst the other end of the spectrum should feature species with high levels of vocal control that are able to modify the individual elements (such as syllables) of a vocalization (Miller et al., 2003).

Vocal control has been investigated in a diversity of species (bats, Smotherman, 2007; budgerigars, Osmanski & Dooling, 2009; anurans, Cunnington & Fahrig, 2010; pinnipeds, Torres Borda et al., 2021; monkeys, Hage et al., 2013, 2016; Hage & Nieder, 2015; Gavrilov & Nieder, 2021), but most studies have focused on songbirds and the neuronal circuitry underpinning their songs (Brenowitz, 2004; Keller & Hahnloser, 2009; Konishi, 1985). Some species can change the duration or the average pitch of their vocalizations, and also the timing, number, arrangement, and structure of syllables or other basic units (Brumm, 2006; Carlson et al., 2020; Slabbekoorn & Peet, 2003; Templeton et al., 2005; Villain et al., 2016). They can also modulate the intensity of the vocalization, which has been shown in several bird species (The Lombard effect, i.e., a modification of the vocal output to compensate for surrounding noise) (Brumm et al., 2009; Brumm & Todt, 2002; Luo et al., 2018; Manabe et al., 1998). Previous researchers have also used interruptibility experiments, which detect the ability of an individual to interrupt its song in response to an external acoustic stimulus. Heuglin's robins (Todt, 1971), blackbirds (Todt, 1975), nightingales (Hultsch & Todt, 1982), Bengalese finches (Seki et al., 2008), and chaffinches (Heymann & Bergmann, 1988) can interrupt their songs upon hearing a noise. Several species can also interrupt their vocalizations in response to a non‐acoustic stimulus (e.g., light flash stimulations). This has been shown in zebra finches (Cynx, 1990) and nightingales (Riebel & Todt, 1997), but also in doves (ten Cate & Ballintijn, 1996), which are not songbirds. Interestingly, the subjects in these experiments often completed the ongoing syllable before stopping (Cynx, 1990). Doves were not always able to interrupt an ongoing vocal element if the flash occurred early during the element (ten Cate & Ballintijn, 1996). These experiments provide some evidence of vocal control and show the limits of this control over the production of vocalizations. In a recent study (Brecht et al., 2019), two crows were experimentally trained to emit a call following the appearance of a Go‐visual cue. They started vocalizing within 2–3 s of the stimulus presentation and did not vocalize when a No‐Go‐cue was shown. The authors argue that crows were capable of volitional control over the onset of their vocalizations (Brecht et al., 2019), as their calls were not triggered by affective (and involuntary) processes. Although volitional control has been studied in monkeys (Coudé et al., 2011; Ghazanfar et al., 2019; Hage et al., 2013), the large number of studies on vocal control has shown very little interest in the volitional aspects of vocalizations in other species, and particularly birds. More generally, the notion remains difficult to demonstrate, as it requires a clear demonstration of the dissociation between emotional and non‐emotional control of the vocalizations.

Still, it could be hypothesized that species with advanced cognitive performances have greater control of their vocal output. Very few studies have assessed the connection between non‐vocal cognitive skills and vocal production in songbirds. A study on song sparrows seems to indicate that the size of the song repertoire may correlate with performances in a detour reaching task (Boogert et al., 2011; but see also DuBois et al., 2018 for opposite results). However, song repertoire size does not correlate with other cognitive measures (Boogert et al., 2011). More generally, intra‐species performances in birds do not always correlate from one cognitive task to another (Boogert et al., 2011). However, one group of species has been extensively studied for their social and physical cognitive skills and shows good and consistent cognitive performances in a large diversity of cognition tests: the corvids (Lefebvre et al., 2004). Corvids are technically songbirds, but they have not received much attention in acoustic studies compared to other species of songbirds. Described as “songbirds without songs” (Fitch & Bugnyar, 2015), they emit a series of vocalizations that are sometimes produced in no particular context (Brown, 1985; Marler, 2004). They have also been anecdotally reported to imitate human voices in the same way that parrots do and can produce duetting behavior (Seed et al., 2007; Kondo et al., 2010; Reber et al., 2016). They have excellent learning, memory, and planning skills, show long‐term vocal recognition of congeners, and have sophisticated social cognitive skills (Boeckle & Bugnyar, 2012; Bugnyar et al., 2016; Emery, 2004; Emery et al., 2007; Güntürkün & Bugnyar, 2016; Raby et al., 2007). They also appear to have the same neural song system as other oscines (Kersten et al., 2021; Wang et al., 2009). We can, thus, expect them to have good control over their vocal production as suggested by the crow study in Brecht et al. (2019).

This study investigates vocal control in another species of corvids, the rook (*Corvus frugilegus*). We had previously observed that certain birds in a captive colony of 14 adult rooks would spontaneously produce long series of different vocal elements (squawks, sneezes, snores, or cackles), sometimes upon hearing loud noise, such as engine noises like passing planes or motorbikes (Video S1). Those vocalizations, which we are currently investigating through other studies, are vocal sequences comprising approximately 15 different vocal elements. The sequences are heterogeneous between individuals. Rooks also often produce these long series of vocalizations during the daily filling of their small bathing pool. To investigate vocal control over these series of vocalizations, we exposed the birds to various durations of water noises and silences during the daily filling of their pool (Video S2). If rooks display vocal control, they should somehow coordinate their vocalizations to this stimulus: showing temporal contingency to the start of the stimulus, but also being able to stop when the stimulus stops. Unlike Brecht et al. (2019), our study concerned long sequences of vocalizations (several seconds) and individuals were not trained to vocalize on cue. Our study, thus, provides complementary data on corvids vocal control with a more ecologically embedded approach.

## 2 MATERIAL AND METHODS

### 2.1 Subjects

The studied group was housed on the CNRS campus of Cronenbourg in Strasbourg, France. Ten of these birds were collected from a wild local colony after they fell from the nest. They were then raised together and handfed by humans for a few weeks until they reached feeding autonomy. After a few months, they became very independent from humans, avoiding contact and staying cohesive with the other members of the group. In 2016, wild birds, collected from hunting traps, integrated the group: one was a 4‐month‐old immature male, and the others were adult females. Group composition changed slightly over the duration of the study (which spanned 4 years, from 2014 to 2018), with 14 birds (9 males and 5 females) in 2014, 16 in 2016 (9 males, 7 females), and 14 in 2017 (8 males and 6 females, Table 1). Only three rooks took part in the experiment described in this study: Kafka, who is one of the highest ranking males, Tom who has an intermediary rank, and Brain who is often the lowest ranking male in the group hierarchy. The rooks were housed in a large outdoor aviary (18  × 6  × 3.5 m) containing wooden perches, platforms, suspended baskets, ropes, vegetation cover, and branches, as well as two small water pools for enrichment and bathing. Individuals were fed daily with a mixture of pellets and fresh products (eggs, yoghurt, and fruit) and had ad libitum access to water. All birds were easily identifiable through colored leg rings.

**TABLE 1:** Individuals in the colony by the end of the study (2017).

Name	Sex	Capture year	Age type
Bashir	M	2013	Adult
Feisty	F	2016	Juvenile
Brain*	M	2006–2007	Adult
Elie	M	2006–2007	Adult
Gigi	F	2013	Adult
Jolene	F	2016	Juvenile
Jonas	F	2006–2007	Adult
Kafka*	M	2006–2007	Adult
Merlin	M	2006–2007	Adult
Noah	M	2006–2007	Adult
Osiris	M	2006–2007	Adult
Pom	F	2013	Adult
Siohban	F	2013	Adult
Tom*	M	2006–2007	Adult

*Note*: The three birds which took part in the experiment are marked by a *.

### 2.2 General procedure

**FIGURE 1:**
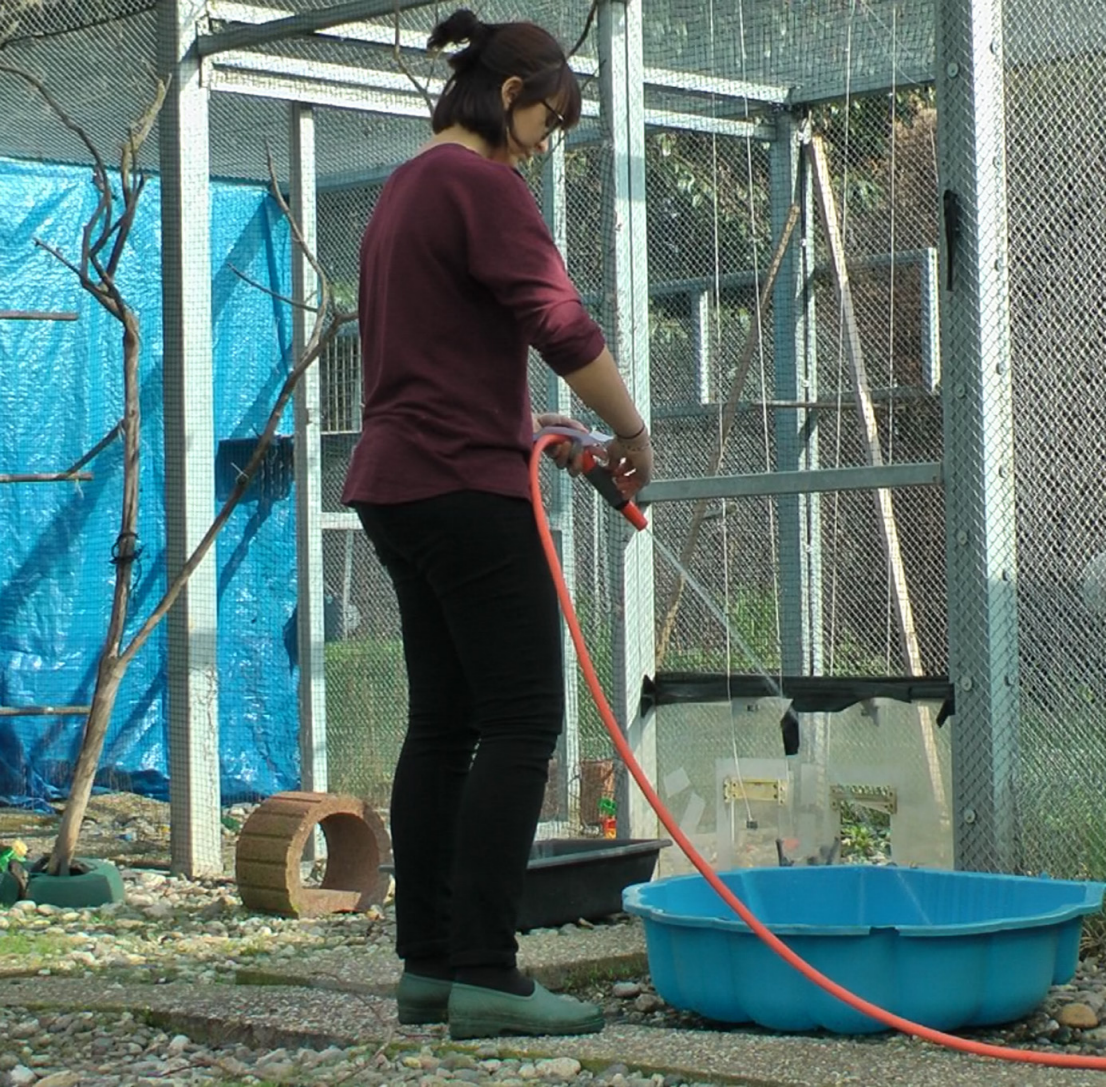
Illustration of the experimental setup.

The tests took place at the normal time of the daily aviary cleaning routine. Thus, one of the bathing pools was first emptied, scrubbed, and then rinsed by the experimenter, as carried out in the usual daily routine. The bathing pool was a small plastic recipient in the form of a seashell (75 × 85 × 25 cm). The cleaning of the pool lasted approximately 30 s to a minute. The main experimenter then stood still, holding a water hose (to fill up the pool), a chronometer, and a list of phase durations for the filling and pause phases (Figure 1). The experimenter was never looking toward the birds to avoid inadvertently providing them with cues.

**FIGURE 2:**
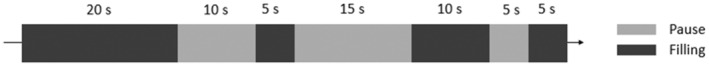
Example of the beginning of a session. Each session started with a 20‐s filling phase.

All test sessions were conducted in a group setting, and all members of the group were free to join the experiment (i.e., vocalize). However, we focused on the first bird who vocalized (hereinafter referred to as the first singer) to investigate potential coordination with the noise. Note that other birds could also vocalized, but given that it was not possible for us to decipher if they were then reacting to the stimulus or to the vocalizations of the first singer, we chose not to record their behavior and focused the camera on the first singer only. Test sessions always started with the first 20‐s phase of water filling of the pool, to signal the beginning of the experiment to the birds (Figure 2). The experimenter then continued the session with alternate phases of fillings, which produced a water noise, and pauses. These phases of filling and pauses will hereafter be referred to as “phases”. Phases of filling and pauses lasted 5, 10, 15, or 20 s.

The durations of the phases following the initial filling were randomly determined before the experiment so that they could not be predicted by the birds. An observer sat quietly in a corner of the aviary without moving, holding a camera, and looking down at the screen of the camera, filming the first bird that vocalized. In some rare circumstances, a bird could stop vocalizing after only a few phases. If another bird then started to vocalize, it became the new focal individual (hereinafter referred to as the second singer) and was subsequently filmed for the whole duration of the session (this occurred only twice). Note that we had no control over the duration of involvement of the birds, as they were free to vocalize or to remain silent at any time during the sessions. However, given that all birds in the enclosure could hear the sound of the water‐filling noise, both primary and secondary singers that vocalized had been exposed to the same sequences of stimuli. Birds were not encouraged to come closer to the pool or vocalize in any way and were not rewarded in any way before, during, or after the session. In many sessions, no birds vocalized (vocalizations occurred in 22 sessions out of 25 in 2014, with sessions 8 and 19 having one primary then one secondary singer; in five sessions out of five in 2015 with only one primary singer; and in only six sessions out of 23 in 2017 with only one primary singer). Sessions including vocalizations were scored using the software The Observer (*Noldus inc*.) with a video framerate of 25 fps and an audio sampling rate of 48 kHz.

During the first part of data collection, from January to May 2014, sessions lasted for 250 s, allowing for five repetitions of each duration for both phases (filling and pause). Later (May 2016), the birds only started to show interest in the last minutes of the tests. For the sessions conducted in May 2016 and from May to June 2017, we, therefore, decided to double the session length in order to maximize the chances of a bird vocalizing. Each duration was then presented 10 times. In total, 53 sessions were conducted (25 in 2014, five in 2016, and 23 in 2017).

Whenever a bird vocalized, we scored the duration, starting, and stopping points of each series of vocalizations and phases, as well as the phase type (i.e., filling or pause). The sound of the filling of the bathing pool partially masked the vocalizations. We, therefore, also used the body movements of rooks (typical and easily identifiable, Video S2) to support the identification of vocalizations. Those visual and sound cues were used to identify the onsets and offsets of the vocalizations, the time precision unit being of 40 milliseconds (the videos were recorded at 25 fps). We also reported whether other birds (and which one) had produced a series of vocalization outside the focus of the camera.

We also analyzed “natural” series of vocalizations (Video S1) as a means of comparison with those produced in the context of the experiment. Those series of vocalizations comprised at least two different vocalization types repeated several times. They were recorded over a period of 5 years (2013–2018) and occurred outside of any experimental context. We analyzed the length of 49 series for Brain and 40 for Kafka using the software Audacity. Several series of vocalizations could be produced in a row but they were always separated by at least 2 s, a duration well above the median duration of inter‐vocalization intervals (median_Kafka_ = 0.6 s, median_Brain_ = 0.5 s). On average, each of Brain's “natural” series of vocalizations lasted 10.8 s without pause, while each of Kafka's lasted 12.5 s.

### 2.3 Statistics

We first examined the proportion of vocalizing events that occurred during fillings versus pauses using a binomial exact test (null hypothesis set at 50%, “stats” package in R) for the singers. As the birds vocalized at different rates, these proportion tests were conducted per individual. Second, we hypothesized that if birds vocalized in temporal contingency to the stimulus, the duration of their series of vocalizations should depend on the duration of the filling stimulus, rather than on the duration of the pause. To investigate this question, we ran a linear mixed effect model to test the effect of the interaction of the “duration of phases” and the type of phase (filling or pauses) as fixed effects on the “duration of vocalisations” as the response variable, with the “session” variable used as a random factor (Function “lmer” in R package lme4 v.1.1‐13, Bates et al., 2015). We evaluated the statistical significance of our model compared to a null model lacking all set of tested predictors using a likelihood ratio test (function “ANOVA”, package “stats” in R). Post‐hoc analyses were conducted using a Bonferroni correction (function “lsmeans”, in R package LSmeans, Lenth, 2016). It must be noted here that the duration of each phase depended on the manual activation of the hose by the experimenter, meaning that phases could be slightly longer or shorter than the intended duration (93.8% of the filling phases were of the intended duration ± 2 s, 95.6% of the pause phases were of the intended duration ± 2 s). For this reason, the “duration of phases” variable is not implemented as a categorical variable in the analysis, but as a continuous variable with the exact durations. Adopting the same criterion as that used by Brecht et al. (2019), advanced vocal control also implies that individuals should, respectively, start and stop vocalizing within 3 s after the beginning and the end of the filling stimulus. We report the median latency of starting and stopping vocalizing after the beginning and the end of the stimulus, respectively.

Finally, we also used a linear mixed effect model to investigate whether the “duration of the vocalisation” (fixed effect) had an effect on the “latencies to stop vocalising” after the end of the filling phase (response variable), with the “session” variable used as a random factor. This model was also compared to a null model. The alpha level was set to 0.05.

## 3 RESULTS

A total of 53 sessions were conducted. Three male adult birds vocalized during these sessions as main singers (either first or second singer): Brain in 22 sessions, Kafka in 10 sessions, and Tom in 3 sessions. Analyses were not conducted on the whole group because only those three birds took part in the experiment as first or second singer. Note, however, that three other males occasionally vocalized during the experiment (Ellie: thrice, Merlin: once, and Noah: once), but never as first or even second singer. Thus, they were not recorded. In addition, Tom's data were not analyzed due to a very low number of events. Data are first presented for Brain then for Kafka.

**FIGURE 3:**
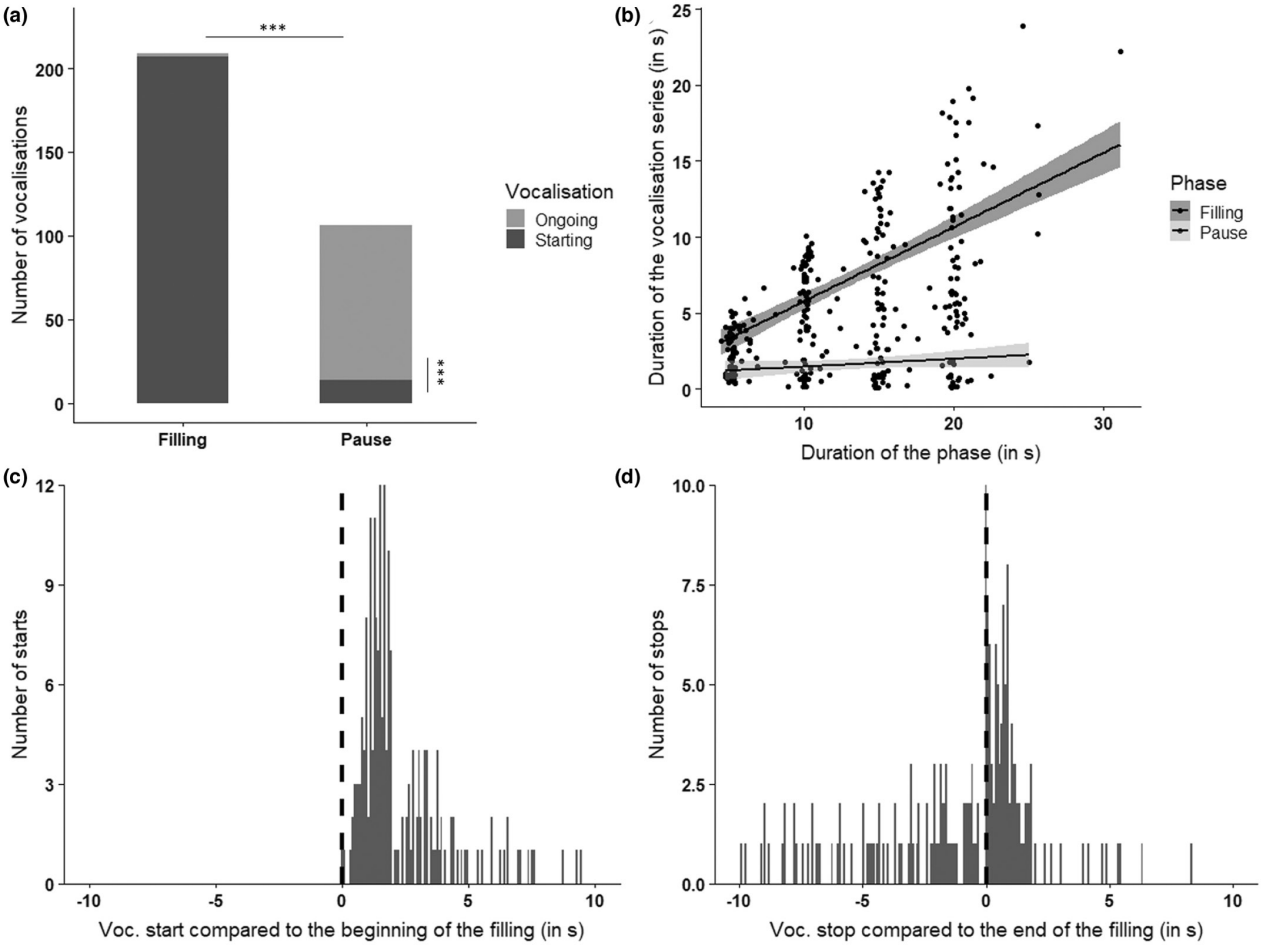
Summary of the results for Brain. (a) Distribution of the series of vocalizations during pauses or fillings of the pool, whether a series of vocalizations started during a phase or was the continuation of a previous series of vocalizations (***p‐value < .001, exact binomial test). (b) Results of the linear mixed model on the effect of the duration and type of phase on the duration of vocalizations. (c) Distribution of the latencies of vocalization outsets compared to the beginning of the filling stimulus. (d) Distribution of the latencies of vocalization endings compared to the end of the filling stimulus.

Brain vocalized significantly more often during fillings than pauses (209 vs. 106 cases, respectively, two‐tailed exact binomial test, *p* < .001). Note that most of the vocalizations recorded during pauses were the continuation (and ending) of the series of vocalizations he had initiated during the preceding filling phase(s) (in 86.9% of cases, exact binomial test, *p* < .001) (Figure 3a). In 93.2% of the cases, he started vocalizing during a filling interval. In three cases, his vocalization started at one filling phase covered the pause, and went on or ended at the next filling phase. If we look at the number of correct responses, that is, only vocalizing during a filling phase or starting vocalizing during a filling phase and stopping shortly after the end of the filling (i.e., when the following pause began), we observe that Brain produced 202 correct responses out of a total of 219 possible responses, thus only a 7.8% error rate. Brain never made more than two errors per session: between the moment the bird started to participate and the moment it stopped, no errors occurred in 11 of the 22 sessions, only one error occurred in five sessions, and two errors were observed in six sessions. A linear mixed effect model indicated a significant effect of the interaction between the variables “duration of phase” and “type of phase” (β = 0.4, T = 6.9, *p* < .001; AIC_model_ = 1586.7, AIC_null model_ = 1869.9, *p* < .001). The duration of vocalizations was more affected by the duration of the filling phases than by the duration of the pauses (β = 5.77; T = 16.7; *p* < .001, Figure 3b). Vocalizations that continued non‐stop over multiple filling phases were excluded from this particular analysis, as well as vocalizations that occurred only during pause phases (which make up 7.3% of the vocalizations by Brain). Brain started vocalizing with a median latency of 1.7 s after the beginning of the stimulus (Figure 3c), and 70.0% of the onsets of his vocalizations occurred within 3 s of the water starting to flow. When he stopped vocalizing after the end of the stimulus (50% of the endings), he did so with a median latency of 0.8 s after the end of the stimulus (Figure 3d), and 91.3% of those endings occurred within 3 s of the water ceasing to flow. A second linear mixed effect model also indicated that the longer Brain had been vocalizing, the longer it took him to stop doing so once the filling stimulus had stopped (β = 0.1, T = 3.3, *p* < .01; AIC_model_ = 341.3, AIC_null model_ = 349.8, *p* < .01).

**FIGURE 4:**
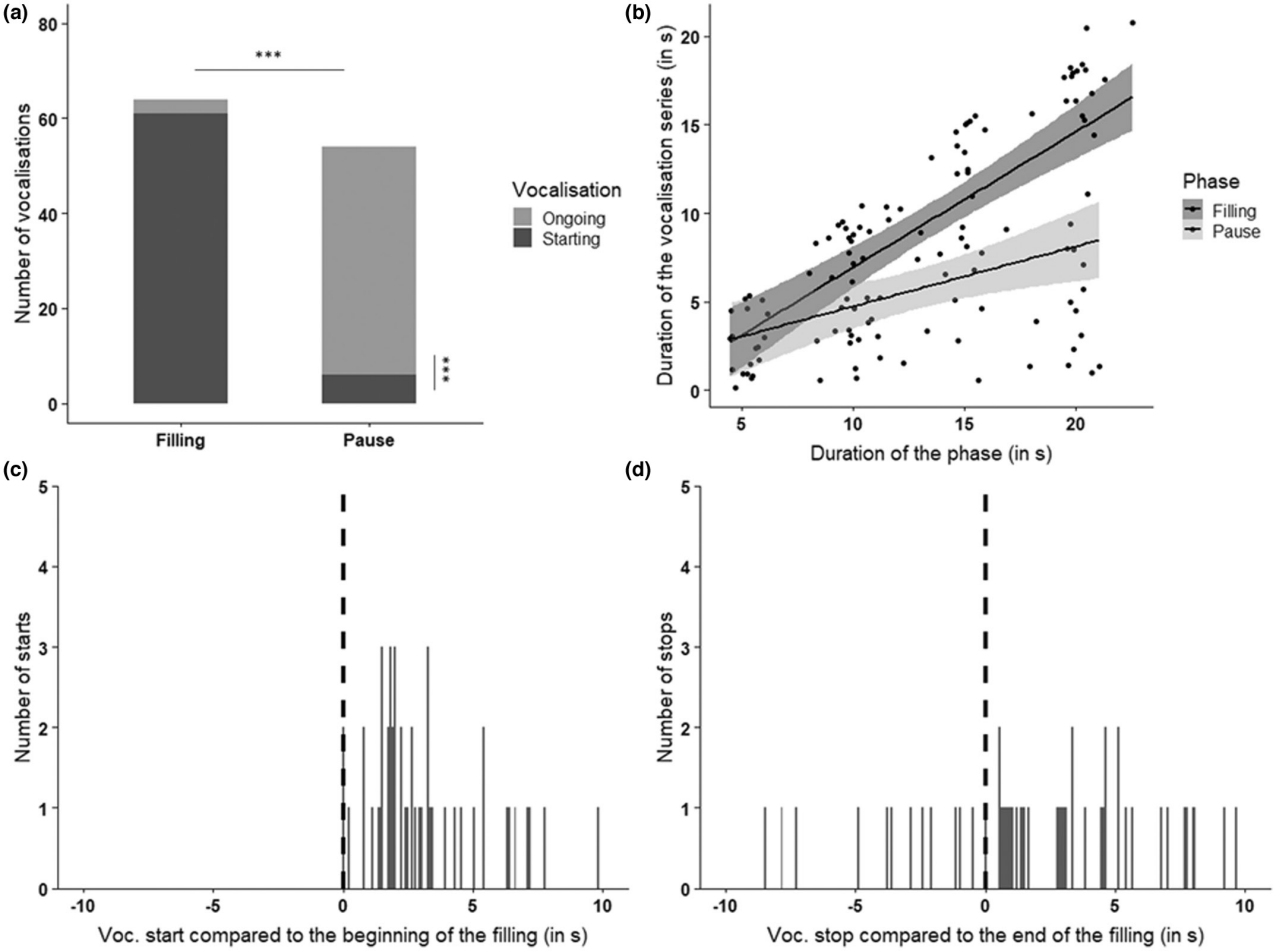
Summary of the results for Kafka. (a) Distribution of the series of vocalizations during pauses or fillings of the pool, whether a series of vocalizations started during a phase or was the continuation of a previous series of vocalizations (***p‐Value < .001, exact binomial test). (b) Results of the linear mixed model on the effect of the duration and type of phase on the duration of vocalizations. (c) Distribution of the latencies of vocalization outsets compared to the beginning of the filling stimulus. (d) Distribution of the latencies of vocalization endings compared to the end of the filling stimulus.

Kafka did not vocalize more often during fillings than during pauses (64 vs. 54 cases, respectively, exact binomial test, *p* = .4). However, 88.1% of Kafka's vocalization series were initiated during a filling phase, and most of the vocalizations recorded during pauses were the continuation (and ending) of a series of vocalizations that had started during the preceding filling phase(s) (in 87% of the cases, exact binomial test, *p* < .001) (Figure 4a). In nine cases, his vocalization started at one filling phase, covered the pause, and went on or ended at the next filling phase. If we look at the number of correct responses, that is, vocalizing only during a filling phase or starting vocalizing during a filling phase and stopping shortly after the end of the phase (i.e., at the following pause), Kafka produced 46 correct responses out of a possible 59 (22.03% error rate). Two of the 10 sessions contained no errors, four contained only one error, and the four remaining sessions contained three errors at the most. The linear mixed effect model indicates that the duration of vocalization series increased with the length of the phases (β = 0.3, T = 3.2, *p* < .01). There was also a significant interaction between the “duration of phase” and “type of phase” variables (β = 0.4, T = 3.0, *p* < .01; AIC_model_ = 741.8, AIC_null model_ = 669.5, *p* < .001). The duration of vocalizations was more influenced by the “duration of filling” phase than by the duration of the pause (β = 3.55; T = 4.8; *p* < .001, Figure 4b). This analysis excludes vocalizations that continued non‐stop over multiple filling phases, that is, 21.7% of the vocalizations. Kafka started vocalizing with a median latency of 2.6 s after the beginning of the filling and 56.9% of his vocalization onsets were within the 3‐s criterion (Figure 4c). When he stopped vocalizing after the end of the stimulus (77.8% of his endings), he did so with a median latency of 3.6 s, and 40.5% of his vocalization stops were within the 3‐s criterion (Figure 4d). Vocalizing duration also had an effect on the latency to stop vocalizing: the longer Kafka had been vocalizing, the longer it took him to stop doing so once the filling stimulus had stopped (β = 0.1, T = 2.3, *p* = .03; AIC_model_ = 225.9, AIC_null model_ = 229.1, *p* = .02).

## 4 DISCUSSION

At the group level, most birds did not react to the stimulus. However, on frequent occasions, two rooks were able to adjust roughly (within 3 s) the timing of their vocalizations to the sound of the water hose filling their bathing pool. These two birds showed good temporal alignment with the external acoustic stimulus, as they generally started to vocalize during the filling phases and stopped at the pauses. They were influenced by the duration of the stimulus: the longer the filling stimulus, the longer the series of vocalizations. The two subjects could also stop vocalizing within 3 s of the end of the filling phases, which was particularly frequent in one bird. This could mean that rooks are able to show good vocal control over the onset and ending of their vocal outputs. Below, we discuss their vocal control abilities and how they can be compared to the volitional control detected in crows by Brecht et al. (2019).

Brecht et al. (2019) established three criteria to demonstrate volitional control. First, the stimulus used needs to be neutral. This means that it should have no particular value or emotional valence. Alarm calls or food calls, for example, are loaded with meaning and may trigger an emotional response over which individuals of most species have little or no control. The stimulus used by Brecht et al. (2019) was a visual signal that had no particular value for the birds at the start of their experiment. The birds learned about its value by operant conditioning, which reduced the risk of attributing an affective value to the stimulus, and their response was a learned vocalization that is not part of their standard repertoire. Our study differs strongly from Brecht et al. (2019), as we did not use any operant conditioning procedure, and the stimulus was never associated with food. Contrary to them, we cannot guarantee that the stimulus was deprived of affective meaning. Rooks sang spontaneously. The filling of the pool may have some relevance for the birds, such as an anticipated pleasure to bathe, although they were never observed bathing directly after the experiment. However, we think it unlikely that their response was a trained response (by associative learning or conditioning). Indeed, few birds vocalized and they did not vocalize at every session. Their response is also not comparable to a typical alarm or food call over which they would have no control. Indeed, they responded with long bouts of undirected songs, rather than with single calls to the stimulus. The undirected songs elicited in our study are generally composed of vocal units that are not necessarily part of their calls' repertoire (Dufour, personal observations), like in most vocal learner species (in budgerigars, for example: Manabe & Dooling, 1997).

We cannot exclude that this species likes to vocalize upon hearing loud noises, or broadband sounds as they often produce these vocalizations upon hearing passing planes. The sound of water would then not be neutral because it has interesting acoustic properties, which stimulate them to vocalize. Vocal responses to water noises have been described in other species. Male chaffinches (*Fringilla coelebs*) can produce “rain calls” during the reproductive season; the core function of these calls is still unknown, although they might simply be a mild alarm call and may also play a role in territoriality (Randler & Förschler, 2011). Observations that bear similarities to our rooks' behavior were made by Jane Goodall (1986), who reported rain dances and waterfall displays in chimpanzees: the sound of falling water triggers a response during which the animals pay attention and react to the stimulus (Goodall, 1986). It is very likely that our birds reacted to the acoustic properties of the stimulus. The question is the amount of control involved in these non‐directed songs.

In Brecht et al. (2019), the two additional criteria for volitional control were as follows: temporal contingency and an absence of response in the absence of stimulus or when another stimulus was presented. More precisely, the authors ran a control condition with no‐go trials in which individuals had to refrain from vocalizing when the incorrect cue was shown. Although we could not run a Go‐noGo procedure, our study provides interesting results to discuss temporal contingency, and the capacity to refrain from vocalizing in rooks. Indeed, once stimulated, the birds did not vocalize for random durations but tended to adjust to the duration of the stimulus. Using the three second criterion from Brecht et al. (2019), one bird, Kafka was good at starting just after the beginning of the filling phase (median latency to start 2.6 s), but often failed to stop at the end of this phase (median latency to stop 3.8 s). Continued vocalizations during a pause could mean that the bird knew that a new filling phase would start after the pause, so he saw no reason to stop. This hypothesis might seem speculative, but we cannot exclude this possibility. However, it could also mean that stopping (refraining from vocalizing) was difficult. Indeed, we showed that the longer the birds vocalized, the longer it took them to stop doing so. This may suggest that once the bird had been vocalizing for some time, stopping requires a stronger level of focus and the exertion of a stronger control. The second bird, Brain, was better at adjusting the duration of his vocalizations to the duration of the stimulus, both at the start (median latency to start 1.7 s) and at the stop (median latency to stop 0.8). He performed several perfect sessions in which he vocalized at every single filling phase and stopped at every single pause. In both birds, the duration of the series of vocalizations produced outside of the context of this study was close to 10 s. This means that adjusting over really long (15 or 20 s) or short intervals (5 s) is not explained by their usual way of singing.

As far as motivational aspects are concerned, it might be hard to grasp a reason for this curious and utterly spontaneous vocalizing behavior. This behavior appears reminiscent of song overlapping in countersinging in birds (Logue, 2021) but rooks are not territorial and, here, they vocalized during sessions that occurred outside the reproductive season, with no specific social context involving conspecifics. The vocalizations were also not directed toward an identifiable recipient. The birds were oriented toward the small bathing pool or toward the lawn outside the aviary. Some wild rooks have been seen to perform similar series of vocalizations when located on streetlights or in trees (Dufour, personal observations; Coombs, 1960) so the production of these series of vocalizations by rooks is not an artifact of life in captivity. One interrogation concerns the low number of individuals who could be recorded as the main singer in this study. Only three birds took the role of first or second singer. Over the years, we automatically recorded this vocalizing behavior (in absence of human experimenters). Brain, Kafka, and Tom are the three most frequent singers, which probably explains that they were the first to vocalize and become the primary singers on which we focused the camera. Note also that rooks tend to sing on their own, they usually go away from the colony, perch high, and produce these series of vocalizations that are not directed toward conspecifics (Coombs, 1960). It is possible that when a rook vocalize in such a way, others tend to listen rather than sing along. This would explain why in most cases only one bird was singing at a time. It also explains why it was difficult to record additional birds as main singers.

The vocal repertoire of rooks is very unlike those found in most birdsong. Their calls are harsh, rarely tonal, and mostly do not have harmonics. Still, it would probably be a mistake to consider that their calls require less control or modulations than those of any other songbird species. Corvid vocalizations need to be studied in greater details and compared to other songbirds. Interruptibility experiments were used to determine the basic acoustic units of songbirds' vocalizations, for example, syllables in zebra finches (Cynx, 1990). Our study works as a kind of “reverse” interruptibility experiment, in the sense that the vocalizations were started upon the beginning of the stimulus and stopped upon its end. Notice that most endings of the vocal output occurred within 2 s of the end of a filling phase. This latency is consistent with the one displayed by some songbirds in interruptibility experiments (Riebel & Todt, 1997).

Our hypothesis was that rooks, which have demonstrated good performances and cognitive flexibility in many social and physical cognition tasks, should be able to show vocal control. We acknowledge that our experiment is less controlled than that carried out in crows and that we had no control over the willingness of the birds to come and participate. Whether our study allows addressing the three criteria from Brecht et al. (2019) is also debatable. Despite strong inter‐individual differences in the willingness to vocalize in this study (which resulted in a sample size of only two birds) the birds who participated showed rather good vocal control. One bird in particular fulfilled the temporal contingency criterion, and further studies are needed to explore their vocal control aptitudes. Vocal control should also be investigated in other songbird and non‐songbird species, in connection to general cognitive skills. This work also calls for further studies of the acoustic properties and vocal flexibility of corvid vocalizations, because these “songbirds without songs” may have much to reveal about vocal complexity after all.

## AUTHOR CONTRIBUTIONS

**Maelan Tomasek:** Conceptualization (equal); formal analysis (equal); investigation (equal); methodology (equal); visualization (equal); writing – original draft (equal). **Andrea Ravignani:** Investigation (supporting); supervision (supporting); writing – review and editing (supporting). **Palmyre Boucherie:** Investigation (equal); writing – review and editing (equal). **Sophie Van Meyel:** Investigation (equal); writing – review and editing (equal). **Valerie Dufour:** Conceptualization (equal); formal analysis (equal); funding acquisition (equal); investigation (equal); methodology (equal); project administration (equal); resources (equal); supervision (lead); visualization (equal); writing – original draft (equal); writing – review and editing (equal).

## FUNDING INFORMATION

None.

## CONFLICT OF INTEREST STATEMENT

None.

## Supporting information


Data S1.

Supporting information S1.

Video S1.

Video S2.

## Data Availability

The data that support the findings of this study are available in the supplementary material of this article as .csv file. Additional information can be obtained from the corresponding author VD.
