# Supplementary material for: Spontaneous vocal coordination of vocalizations to water noise in rooks (Corvus frugilegus): An exploratory study
Source: Ecol Evol. 2023 Feb 16;13(2):e9791. doi: 10.1002/ece3.9791 (PMC9936512; doi:10.1002/ece3.9791)
Supplement: Supplementary file 2 — Supporting information S1. [file ECE3-13-e9791-s003.docx]

Supplementary video 1: "Spontaneous vocalisations in a rook"

Supplementary video2  : "Brain adjusting his vocalisations to the the water noise"
